# A *de novo* missense mutation of *FGFR2* causes facial dysplasia syndrome in Holstein cattle

**DOI:** 10.1186/s12863-017-0541-3

**Published:** 2017-08-02

**Authors:** Jørgen S. Agerholm, Fintan J. McEvoy, Steffen Heegaard, Carole Charlier, Vidhya Jagannathan, Cord Drögemüller

**Affiliations:** 1 Department of Clinical Veterinary Sciences, Faculty of Health and Medical Sciences, University of Copenhagen, Dyrlægevej 16, 1870 Frederiksberg C, DK Denmark; 2 Department of Pathology, Rigshospitalet, University of Copenhagen, Frederik V’s Vej 11, 2100 Copenhagen Ø, DK Denmark; 3 Department of Ophthalmology, Rigshospitalet, University of Copenhagen, Blegdamsvej 9, 2100 Copenhagen Ø, DK Denmark; 4 Unit of Animal Genomics, GIGA-R & Faculty of Veterinary Medicine, University of Liège, 4000 Liège, Belgium; 5 Institute of Genetics, Vetsuisse Faculty, University of Bern, Bremgartenstrasse 109a, 3001 Bern, Switzerland

**Keywords:** Bovine, Congenital, Malformation, Rare disease, Hereditary, Fibroblast growth factor receptor 2, Crouzon syndrome, Pfeiffer syndrome

## Abstract

**Background:**

Surveillance for bovine genetic diseases in Denmark identified a hitherto unreported congenital syndrome occurring among progeny of a Holstein sire used for artificial breeding. A genetic aetiology due to a dominant inheritance with incomplete penetrance or a mosaic germline mutation was suspected as all recorded cases were progeny of the same sire. Detailed investigations were performed to characterize the syndrome and to reveal its cause.

**Results:**

Seven malformed calves were submitted examination. All cases shared a common morphology with the most striking lesions being severe facial dysplasia and complete prolapse of the eyes. Consequently the syndrome was named facial dysplasia syndrome (FDS). Furthermore, extensive brain malformations, including microencephaly, hydrocephalus, lobation of the cerebral hemispheres and compression of the brain were present. Subsequent data analysis of progeny of the sire revealed that around 0.5% of his offspring suffered from FDS.

High density single nucleotide polymorphism (SNP) genotyping data of the seven cases and their parents were used to map the defect in the bovine genome. Significant genetic linkage was obtained for three regions, including chromosome 26 where whole genome sequencing of a case-parent trio revealed two *de novo* variants perfectly associated with the disease: an intronic SNP in the *DMBT1* gene and a single non-synonymous variant in the *FGFR2* gene. This *FGFR2* missense variant (c.927G>T) affects a gene encoding a member of the fibroblast growth factor receptor family, where amino acid sequence is highly conserved between members and across species. It is predicted to change an evolutionary conserved tryptophan into a cysteine residue (p.Trp309Cys). Both variant alleles were proven to result from *de novo* mutation events in the germline of the sire.

**Conclusions:**

FDS is a novel genetic disorder of Holstein cattle. Mutations in the human *FGFR2* gene are associated with various dominant inherited craniofacial dysostosis syndromes*.* Given the phenotypic similarities in FDS affected calves, the genetic mapping and absence of further high impact variants in the critical genome regions, it is highly likely that the missense mutation in the *FGFR2* gene caused the FDS phenotype in a dominant mode of inheritance.

**Electronic supplementary material:**

The online version of this article (doi:10.1186/s12863-017-0541-3) contains supplementary material, which is available to authorized users.

## Background

During the last 25 years, advances in veterinary genetics have significantly improved the prospects for studies into the molecular causes of congenital anomalies in cattle [1] and in the last decade, the availability of genome-wide single nucleotide polymorphism (SNP) arrays combined with the typical structure of livestock populations have markedly accelerated the positional identification of recessive mutations that cause inherited defects in cattle [2]. After the establishment of a bovine reference genome sequence [3], the advent of next-generation sequencing has improved the possibilities enormously [4]. For example, the identification of dominant acting *de novo* mutations, which are a risk in cattle breeding, became feasible due to efficient re-sequencing of whole cattle genomes [5]. Online Mendelian Inheritance in Animals (OMIA), a catalogue of inherited disorders and associated genes in domestic animals, reports more than 500 inherited phenotypes in cattle [6]. Currently, the causal gene mutations for approximately a quarter of these bovine phenotypes have been determined.

A detailed surveillance program for monitoring congenital syndromes in cattle is of great value, especially to identify new phenotypes [5, 7, 8]. From late 2015, a series of cases of a hitherto unrecognized congenital syndrome occurred among the progeny of a Danish Holstein sire. Seven cases were reported to the Danish bovine genetic disease program [9] and materials were submitted for necropsy and genetic analysis. Here we report the phenotype of this novel bovine anomaly, designated as facial dysplasia syndrome (FDS) (OMIA 002090-9913), and efforts to unravel its genetic cause.

## Methods

### Animals

Five malformed Danish Holstein calves (cases 1-5) and the head of additional two cases (cases 6-7) were submitted for examination. Cases 1-5 and 7 were females while the sex of case 6 was not recorded (Additional file 1). All calves were registered as offspring of the Holstein bull VH Myles (DK256738) but were born in different herds. Ethylenediaminetraacetic acid (EDTA) stabilized blood from the dams of the seven examined cases and from a further two cows that had given birth to photo documented malformed cases was available. One of these cases originated from Sweden. Semen and EDTA stabilized blood of VH Myles was obtained as was semen of seven ancestral sires to either VH Myles or dams of affected calves. Finally, EDTA stabilized blood samples from 15 phenotypically normal daughters of VH Myles and their dams were collected.

### Post mortem examinations

The malformed calves were submitted to the University of Copenhagen for full or partial necropsy, (cases 1-5 and 6-7, respectively). The skull was either opened for removal of the brain *in toto* or the head was frozen at -20 °C and then sectioned longitudinally through the midline to visualize the brain and to obtain high quality radiographs.

Specimens of heart, lung, liver, spleen, kidneys, adrenal glands, thymus, thyroid gland, skeletal muscle and spinal cord were fixed in 10% neutral buffered formalin for histology. The brain (cases 1, 3 and 4) and eyes (cases 1-3) were formalin fixed *in toto*. The tissues were processed by routine methods, embedded in paraffin, sectioned at 2-3 μm and stained with haematoxylin and eosin.

To illustrate bone malformations, the head of three calves (cases 1-3) underwent computed tomography (CT) scanning as previously described [5]. Lateral radiographs were obtained from four calves (cases 1, 3-5).

### Breeding analysis

The pedigrees of the parents of affected calves were analysed for inbreeding loops and shared ancestors.

Data on offspring of VH Myles born in Denmark from his first use until May 31, 2016 were obtained from the Danish Cattle Database. The data included offspring recorded as “defective”, “stillborn”, “dead within 24 hours”, “dead after 24 hours” and “still alive.” A letter including a photo of a FDS phenotype was mailed to all owners of offspring recorded as defective. These were subsequently contacted by phone to determine whether the calf recorded as defective actually suffered from this syndrome. The prevalence of defective offspring in the complete dataset was then calculated.

Furthermore, a subset of the most recently born 500 calves in the dataset, i.e. from May 31, 2016 and backwards was selected for a more detailed study. A questionnaire was constructed and mailed to owners of offspring recorded as “defective”, “stillborn” or “dead within 24 hours.” The questionnaire included a photo of a FDS case, the ear tag number of the dam of the calf and the date of delivery. The owners were then contacted by phone to assess if the offspring had suffered from FDS. The prevalence of the syndrome in the subpopulation was calculated.

The length of the gestation period for FDS affected calves and for all live born calves in the dataset was obtained and compared using the Welch Two-sample t-test.

### Genetic analysis

Genomic DNA was extracted from blood samples of 31 animals (the seven FDS cases, eight normal daughters of VH Myles, their respective dams and the sire VH Myles) and used for genotyping with the GGP HD-150K BeadChip (Illumina), including 139,376 evenly distributed SNPs, at Geneseek (Lincoln, NE, USA). Targeted genotyping of the candidate causative variant was done on DNA of the 31 animals and on genomic DNA obtained from blood of additional seven normal offspring, their dams, semen of VH Myles and of seven sires occurring in the pedigrees of FDS cases.

PLINK software [10] was used for pruning of SNP genotype data the identification of extended homozygous regions with allele sharing across cases as described before [11]. Subsequently the MERLIN v 1.1.2 software [12] was used to carry out non-parametric linkage analysis as described previously [6]. Haplotypes were estimated using MERLIN chromosome-by-chromosome.

### Whole genome re-sequencing and searching for variants

Three individual fragment libraries were prepared from DNA extracted of the blood of FDS case 1 and its parents and subsequently sequenced and analysed as previously described [13]. The genome data corresponding to roughly 15x coverage of the genome was made freely available under accession no. PRJEB18113 [14]. The Delly package [15] was used to detect structural variants in the cleaned BAM files. The snpEFF software [16], together with the UMD3.1/bosTau Ensembl annotation [17], was used to predict the functional effects of all variants detected. In addition, the IGV browser was used for visual inspection of the BAM file [18].

Genotyping of the two candidate variant for FDS was performed as described previously [11] by re-sequencing a 190 bp PCR product for *DMBT1* using forward primer (5-TTTAGGTGGAGAGGCAAACG-3) and a reverse primer (5-TGCATTTATGGGGGTCTCTT-3) and a 202 bp PCR product for *FGFR2* using forward primer (5- AAATCAATGAACCTGCGGCC-3) and a reverse primer (5- GAGGCGATGTGGAGTTTGTC-3).

## Results

### Phenotype

**Fig. 1:**
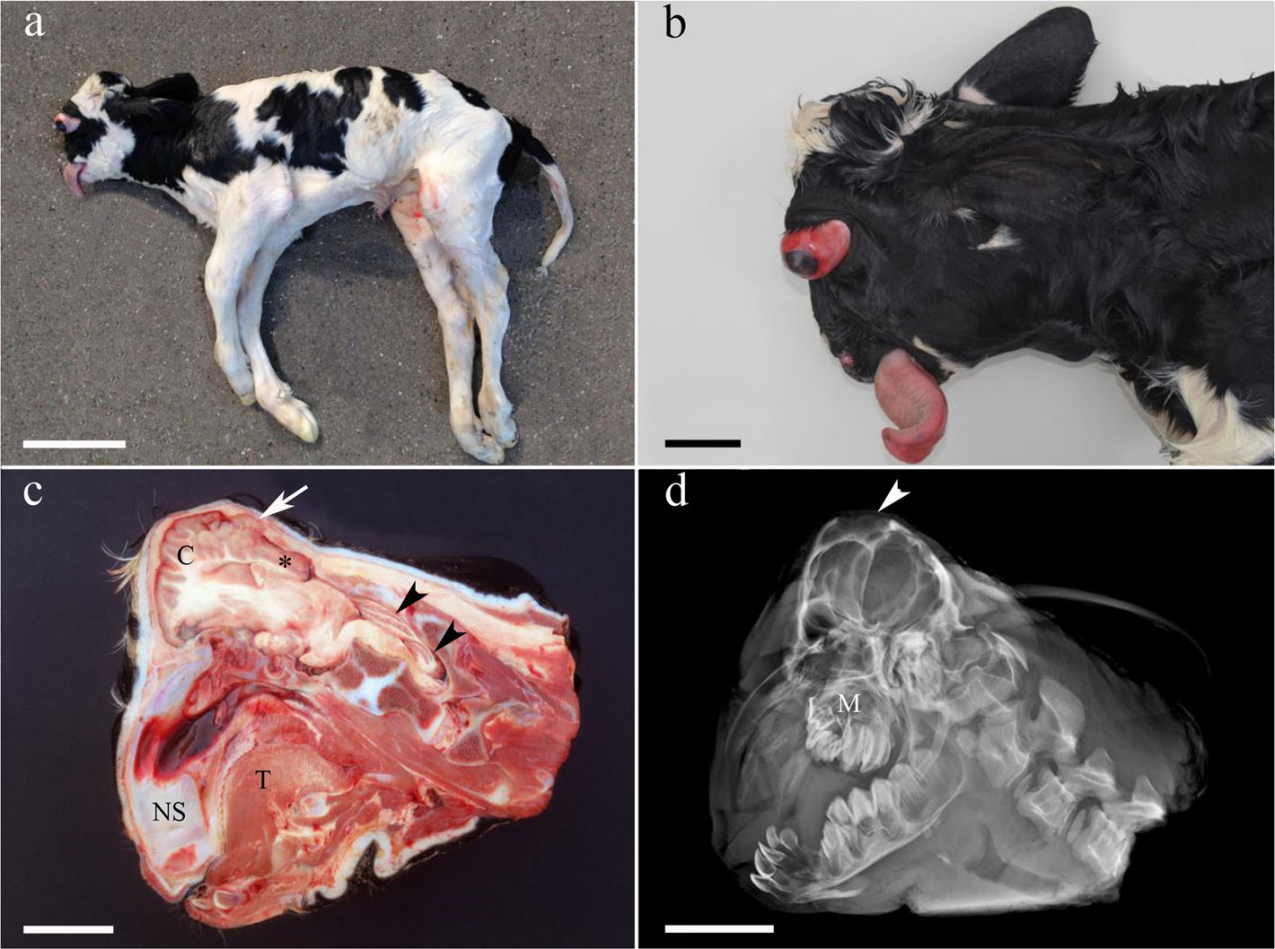
Gross morphology of the facial dysplasia syndrome (FDS). **a**. Overall morphology showing malformation and reduced size of the head and slight bilateral anterior arthogryposis. Bar = 20 cm. **b**. Detail of a FDS case. Notice the prolapse of the eye, dysplasia and ventral deviation of the vicerocranium, protrusion of the tongue and the reduced size of the calvarium. Bar = 5 cm. **c** Longitudinal section of the head. The nasal septum (*NS*) is deviated ventrally. The cerebrum (*C*) is compressed and protrudes through the fontanella (*arrow*) and the occipital lobes are dislocated caudally (*). The cerebellum (*arrowheads*) is compressed and dislocated caudally. *T*: tongue. Bar = 5 cm. **d**. Radiograph of the same specimen as shown in *c*. In addition to the lesions presented in *c*, the abnormal shape and reduced size of the cranial vault is evident. Notice the very thin occipital bones (*arrowhead*) and the severely malformed and undersized maxilla (M). Bar = 5 cm. See Additional file 2 for comparison with a normal calf.

All cases shared a common external morphology predominantly characterized by severe facial dysplasia and bilateral complete prolapse of the eyes (Fig. 1a). Therefore the anomaly was named FDS. Furthermore, all affected calves showed slight anterior bilateral symmetrical arthrogryposis and reduced body weight, e.g. 26.5 kg and 31.5 kg for the two full-term females (cases 2 and 3) compared to normally around 39.6 kg for normal females delivered at term.

The overall size of the head was reduced (microcephaly) (Fig. 1a,b). The vicerocranium showed severe dysplasia with shortening and ventral deviation of the nasal structures, micrognatia superior, shortened mandibles and protrusion of the tongue (Fig. 1b-d). Palatoschisis was present in case 1 and two cases had gingival hyperplasia in relation to the incisor teeth, which in all cases showed varying degrees of misalignment. The eyes demonstrated bilateral symmetrical prolapse, extending several centimetres from the orbits and showed an elongated optic nerve (Fig. 1b). Both orbits were normally developed. The calvarium was of reduced size and form with the anterior part protruding and the posterior part being flattened and prolonged caudally. The fontanel was irregular, open and of increased size. Parts of the frontal bones were of reduced thickness and in some places, the brain was only covered by a thin connective tissue membrane, subcutaneous tissue and skin (Fig. 1c,d). For comparison, the head of a FDS case and a normally developed calf is included as Additional file 2.

**Fig. 2:**
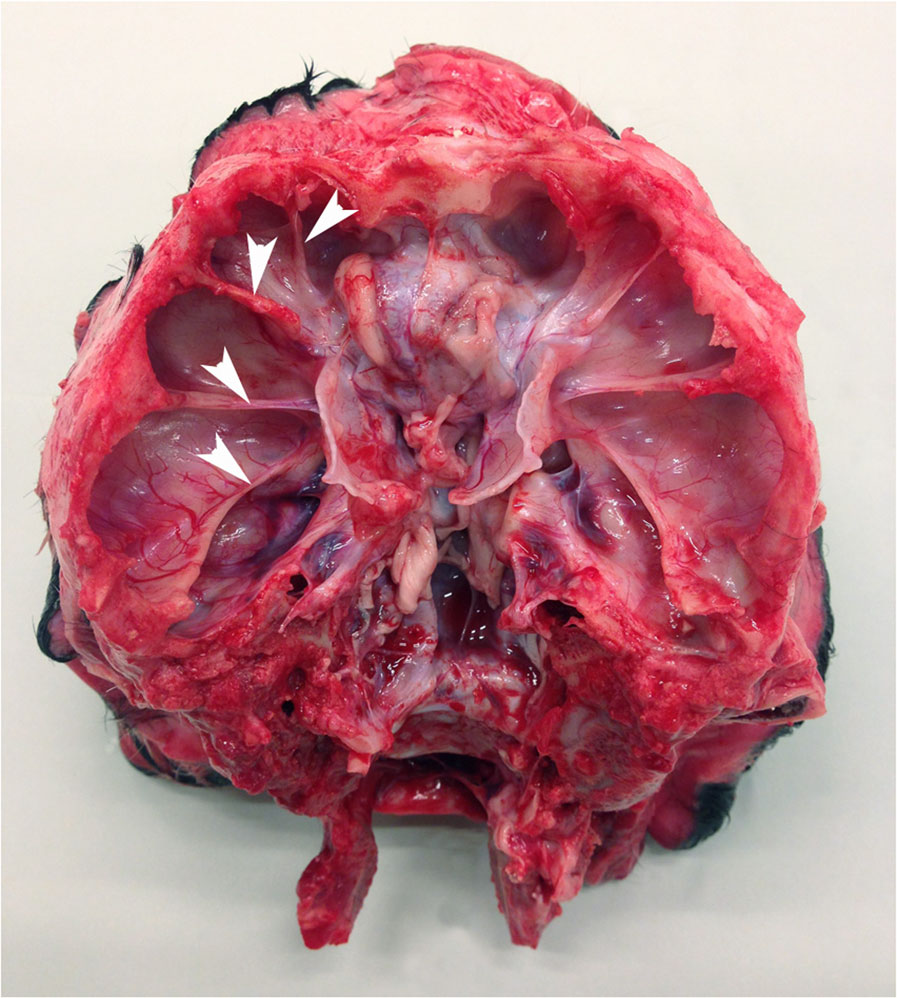
Gross morphology of the cranial vault. Bilateral symmetrical bony ridges (indicated by *arrowheads* in the left side) are present inside the cranial vault and cause pathologic lobation of the cerebrum (not visible; see Fig. 3).

**Fig. 3:**
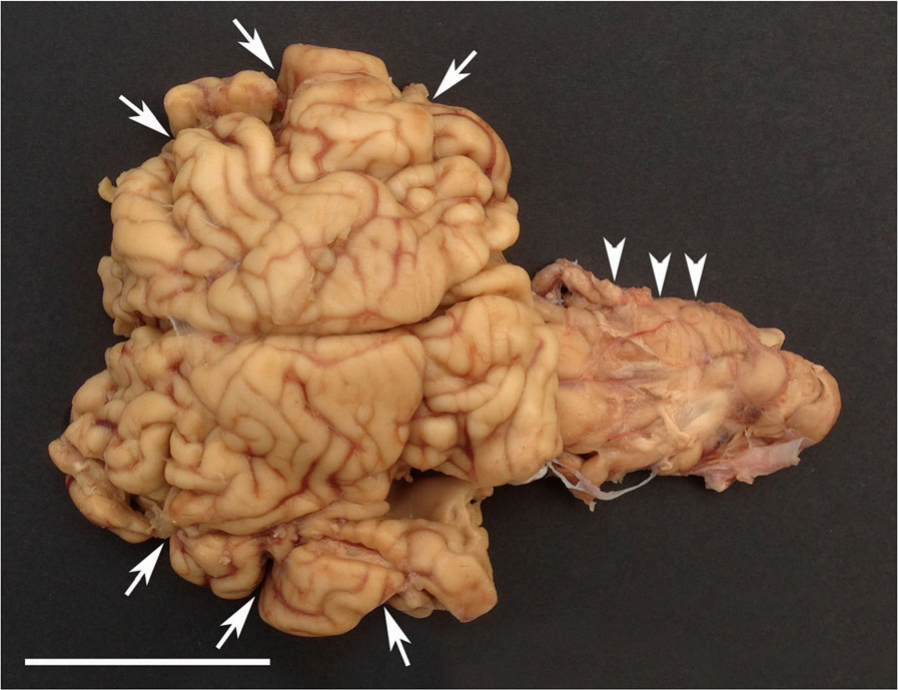
Gross morphology of the brain. The brain is of reduced size and abnormal shape. The cerebral hemispheres are divided into multiple lobes (*arrows*) by sulci developed due to the bony ridges in the cranial vault (not visible; See Fig. 2). The cerebellum is compressed, malformed and longitudinally elongated (*arrowheads*). Formalin fixed specimen. Bar = 5 cm.

**Fig. 4:**
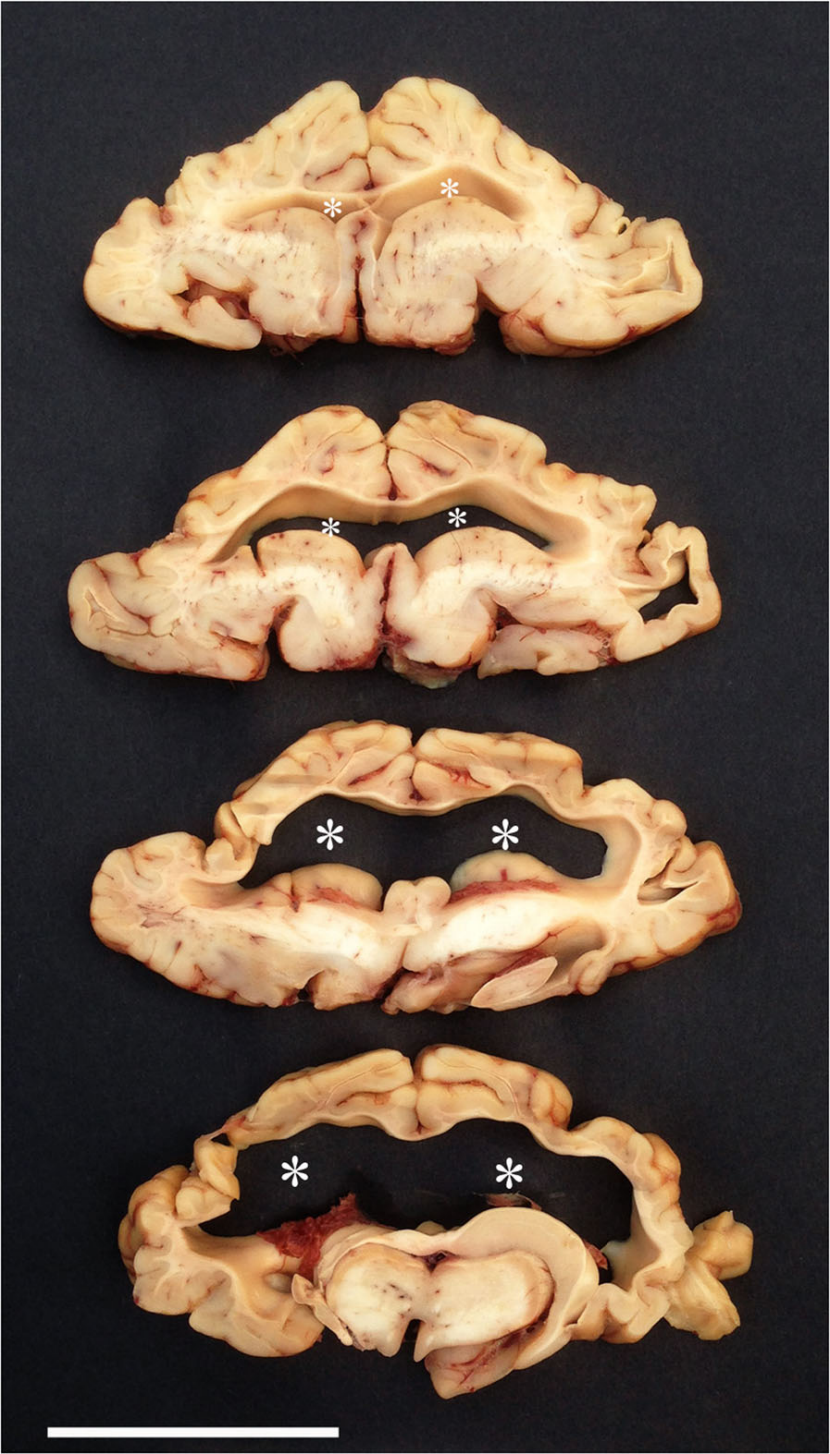
Hydrocephalus. Serial cross sections of the cerebrum showing dilated lateral ventricles (*). Formalin fixed specimen. Bar = 5 cm.

Several bilateral symmetrically developed bone ridges inside the cranial cavity let to lobation of cerebral hemispheres (Figs. 2 and 3) and the reduced and abnormally shaped calvarium was associated with microencephaly and compression of the brain leading to caudal displacement, including herniation through the foramen magnum and abnormal shaping, especially of the occipital lobes, mesencephalon and cerebellum (Fig. 3, Additional file 3). Transverse serial sections of the brain after formalin fixation revealed severe hydrocephalus with distension and abnormally shaping of the entire ventricular system and in some cases also formation of diverticulas extending dorsally from the lateral ventricles to the cerebral surface (Fig. 4, Additional file 3). A lumbar scoliosis was present in case 4.

Histology of the brain was consistent with the gross lesions and characterized by a distended irregularly shaped ventricular system, reduced amount of periventricular neuroparenchyma and abnormally shaped but otherwise morphologically normal structures. The eyes showed slight bilateral cataract but were otherwise normally developed although corneal erosions and neovascularization was present in two cases. Foci of acute haemorrhage were present in several tissues, but otherwise lesions were not observed.

### Breeding analysis

Pedigree analysis of FDS affected calves did not display obvious inbreeding but the analyses showed that their parents often shared several common male ancestors. These sires were usually present nine generations back and occurred in the pedigree of all cases. They belonged to North American breeding lines that have been used extensively in the Holstein breed worldwide and therefore have had a significant impact on the current Danish Holstein population. Two FDS cases were diagnosed in the subpopulation of 500 calves giving a prevalence of 0.40% while 17 calves out of 3639 calves in the complete dataset had suffered from FDS (0.47%).

The length of the gestation period between cows having live-born offspring (n = 3363) *vs.* FDS cases (n = 17) was compared. The mean among the cows with a normal calving was 281 days (SD = 4.9 days), which was significant longer than the mean gestation length of 271 days (SD = 9.1 days) for cows giving birth to a FDS case (*P = *0.0003).

### Genetic analysis

**Fig. 5:**
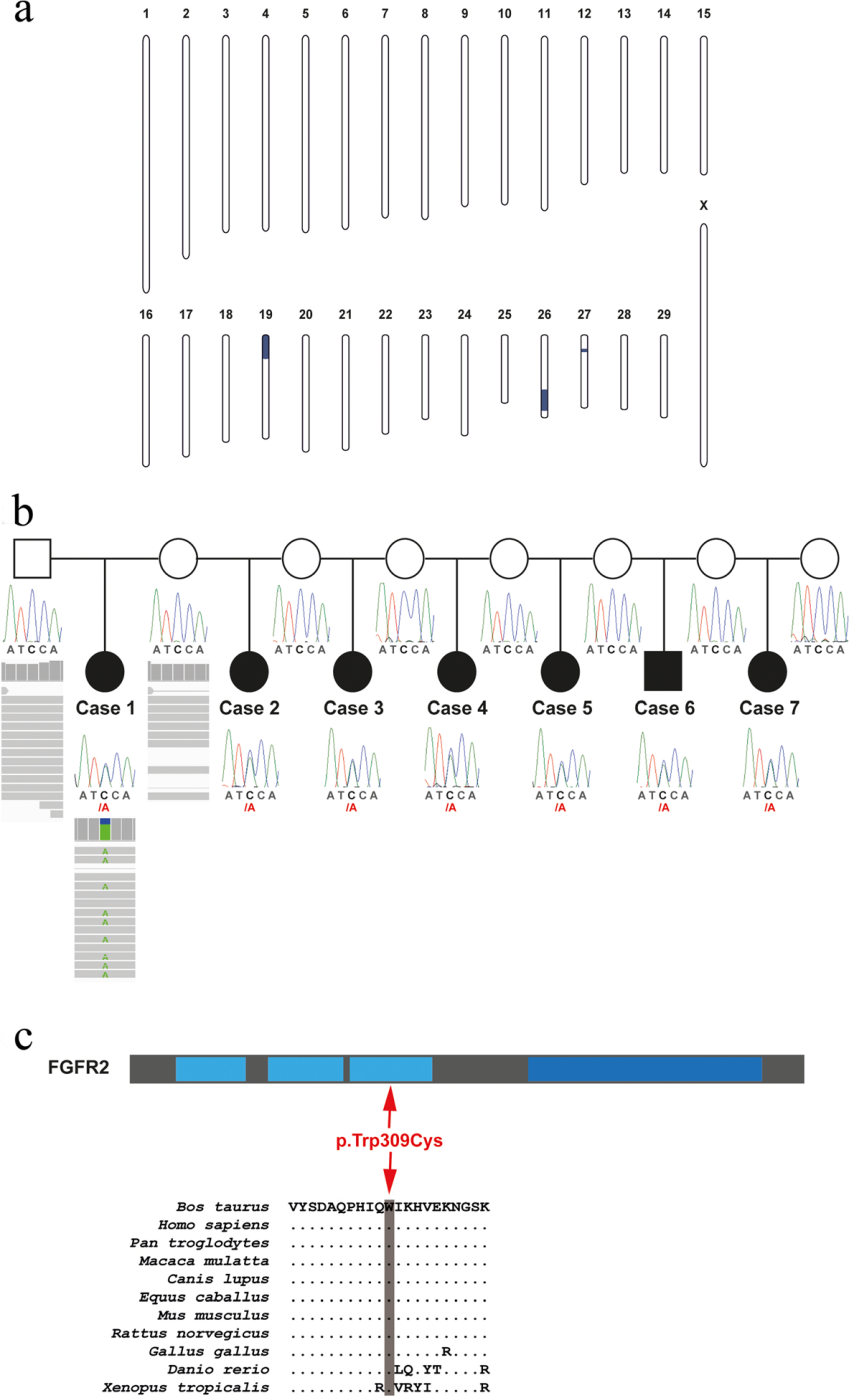
A *de novo* missense mutation of *FGFR2* is perfectly associated with dominant facial dysplasia syndrome (FDS) in a family of Holstein cattle. **a** *.* Non-parametric multipoint linkage analysis for FDS. A total of three significantly linked genome regions are shown in blue. **b** *.* Pedigree drawing and *FGFR2* SNP genotypes. Filled black symbols represent affected calves with FDS, open symbols represent unaffected parents, squares indicate males and circles indicate females. The case-parent trio subjected to whole genome re-sequencing is indicated by IGV screenshots showing the presence of the chromosome 26 g.41'861'956C>A *de novo* variant. Note that the electropherograms presented below the pedigree symbols show that the mutant A allele is present in heterozygous form in FDS affected offspring only. **c** *.* Domain structure of the 840 amino acid FGFR2 protein. The missense variant is positioned in the exon 6 of the *FGFR2* gene. The p.Trp309Cys change affects the third extracellular immunoglobulin-like domain (light blue) in front of a single hydrophobic membrane-spanning segment and a cytoplasmic tyrosine kinase domain (dark blue). The bovine FGFR2 309 tryptophan residue that is substituted by a cysteine residue shows a high degree of conservation across the vertebrate kingdom.

The FDS phenotype occurred in calves having normal parents and all affected calves were sired by VH Myles, which has produced around 99.5% normal offspring. The parents could not be traced back to only a single common ancestor and the phenotype has not been reported before. Identical by descent (IBD) mapping across the seven genotyped FDS cases revealed no single genome interval of homozygosity with shared alleles. Taken together, the segregation pattern of the observed phenotype and homozygosity mapping did not indicate a simple Mendelian recessive inheritance as most likely explanation. Therefore, a non-parametric linkage analysis without assuming a specific segregation model was performed. This analysis detected three contiguous megabase (Mb) sized genomic regions located on three different chromosomes [19, 26, 27] significantly linked with the FDS phenotype at chromosome-wide error probabilities for Z-mean values and LOD scores below 0.01 (Fig. 5a, Additional file 4). Subsequently a haplotype analysis search for disease-linked haplotypes shared across the seven FDS cases was carried out. This analysis detected three paternally inherited haplotypes indicating dominant inheritance: A 12.4 Mb shared haplotype on chromosome 19 (position 0 to 12.4 Mb), an 11.7 Mb shared haplotype on chromosome 26 (position 36.2 to 47.9 Mb), and a 1.6 Mb shared haplotype on chromosome 27 (position 12.6 to 14.2 Mb). In addition, the eight genotyped non-affected daughters of VH Myles were checked for the presence of the shared haplotypes. Each of the three haplotypes was detected in at least one of the normal offspring.

To allow the detection of *de novo* dominant variants, the genomes of a parent-FDS affected offspring trio (case 1) were sequenced. Genome-wide filtering for sequence variants in the whole genome that were present only in the affected calf and absent from the genomes of both parents resulted in 500 private heterozygous sequence variants (Additional file 5). In addition to the SNP and short indel variant calling, larger structural variants like deletions, insertions or duplication were searched but no such variant was exclusively found in the FDS case. Two out of the 500 putative *de novo* variants were located in one of the three previously identified FDS linked genome regions: a missense variant in exon 6 of the *FGFR2* gene (c.927G > T; p.Trp309Cys), and a SNP in the intergenic region of *SPADH2* and *SPADH1*, which acording to the UCSC genome browser [20] is situated in an intron of putative *DMBT1* transcripts (Additional file 6). Both variants were located in the FDS linked genome region on chromosome 26 (g.41'861'956C>A and g.42'862'507G>A). Subsequently, all seven FDS cases, their dams and the sire were genotyped for both variants by Sanger sequencing and for both it was confirmed that the observed alleles in the offspring were due to a *de novo* mutation event, as both parents were homozygous for the wildtype allele (Fig. 5b; Additional file 6). Neither next-generation nor Sanger sequencing showed any presence of the two identified mutations in the sire VH Myles, who clearly carried only the wild type alleles in DNA originating from both blood and semen. The 15 normal offspring of VH Myles were also genotyped as homozygous for the wildtype alleles at both *de novo* variants.

## Discussion

A novel congenital syndrome was recognized in Danish Holstein cattle. The lethal phenotype was designated FDS due to the striking dysplasia of the vicerocranium. The phenotype showed no typical features of already reported anomalies in cattle or other animal species, including known virus-induced malformations in cattle [20]. As the FDS phenotype occurred only within the progeny of a single artificial insemination sire, a heritable aetiology was suspected. The very low frequency of FDS affected offspring (around 0.5%) and the normal phenotypes of the sire and the dams suggest either a recessive disorder, a dominant inheritance with incomplete penetrance or a mosaic germline mutation. A single recessive mutation could be excluded as the affected animals did not show any shared homozygous IBD genome segments. Linkage and haplotype analysis provided evidence for a paternally inherited dominant mutation. Initially, due to the lethal effect, a coding or splice-site variant in a protein coding gene was assumed as causative. By whole-genome re-sequencing of a parent-offspring trio, 500 putative *de novo* sequence variants could be identified. This list contained only a single non-synonymous variant in the *FGFR2* gene; all other variants were located in non-coding intergenic or intronic sequence regions. Two out of the 500 variants, including the *FGFR2* missense variant, were located in one of the three identified linked genome regions and therefore considered as candidate causal mutations. Genotyping all available family members established a perfect association between these two *de novo* variants and the FDS phenotype.

The non-coding variant is located within an intron of a splice isoform of the *DMBT1* gene. This putative tumor suppressor gene plays an important role in human medulloblastomas (OMIM 601969) and so far there is no described *DMBT1* variant in humans associated with a congenital phenotype [21]. Collectively, these data do not support *DMBT1* as the responsible gene for bovine FDS. On the other hand, the non-synonymous variant affects *FGFR2,* a gene known to be associated with severe autosomal dominant syndromes (OMIM 176943) mostly characterized by craniofacial skeletal abnormalities [22]. This gene encodes a member of the fibroblast growth factor receptor family, where amino acid sequence is highly conserved between members and across species. The observed missense variant is predicted to change an evolutionary conserved (invariant in vertebrates) tryptophan into a cysteine residue (Fig. 5c). PolyPhen 2 software based analysis of this amino acid exchange characterized the mutation as highly damaging [23]. Therefore *FGFR2* represents a very good candidate gene for the condition, and the detected *FGFR2* missense variant is much more likely to be responsible for the observed phenotype. Given the linkage analysis results and absence of further variants in the linked genome regions, it strongly suggests that the *FGFR2* missense variant caused the FDS phenotype in a dominant mode of inheritance. This is further corroborated by the fact that this allele resulted from a *de novo* mutation event in the germline of the sire. Probably both *de novo* mutations occurred on the same haplotype and at similar time in a single germ cell that continued to divide. The FDS phenotype was present in less than 0.5% of VH Myles offspring and was associated with the coding variant on chromosome 26. Using standard PCR based methods this variant could not be detected in the sperm cells of VH Myles, which is consistent with the very low frequency of FDS affected offspring. Nonetheless it seems to be that VH Myles represents another example for a germline mosaic in livestock as shown before for Solid Gold for ovine callipyge [24], Campus for porcine myopathy [25], and VH Cadiz Captivo for bovine chondrodysplasia [5]. Interestingly, in human Apert syndrome (OMIM 101200) a paternal origin of the causal *FGFR2* mutation was shown in more than 50 families [26]. VH Myles was culled by the breeding association to avoid occurrence of further FDS cases.

Human patients showing *FGFR2* associated syndromes are characterized by craniosynostosis causing secondary alterations of the facial bones and facial structure. For example the Crouzon syndrome (OMIM 123500) in humans is characterized by craniosynostosis but normal limbs and was initially shown to result from allelic mutations of the third immunoglobulin-like domain of FGFR2 [27]. Subsequently most mutations identified in the human *FGFR2* gene localize to just two exons, encoding the third immunoglobulin-like domain in the extracellular region, resulting in syndromic craniosynostosis including Apert, Crouzon and Pfeiffer syndromes (OMIM 101600) [28]. The mutant residue p.Trp309Cys is also situated in that specific extracellular domain of the ortholog bovine protein (Fig. 5c). Interestingly, the corresponding human FGFR2 tryptophan residue 290 was found identically substituted by a cysteine in patients with severe Pfeiffer clinical features leading to premature death [29, 30]. These patients have “cloverleaf” skull deformity as well as the other typical ocular, hand, and foot anomalies seen in Pfeiffer syndrome. The herein described phenotype of the FDS affected calves differs as it obviously only affects the head without alterations of the limbs. This is in accordance with an obvious phenotypic variability of human patients with other missense mutations at the FGFR2 tryptophan residue 290, which appeared to be a mutational hotspot in the gene [30]. An arginine substitution was observed in classic Crouzon syndrome [31], whereas a substitution of this specific residue to glycine results in an atypically mild form of Crouzon syndrome [32]. A further *FGFR2* missense mutation affecting another residue of the third immunoglobulin-like domain was detected in one Pfeiffer syndrome family in which two members had craniosynostosis without limb anomalies [33].

## Conclusions

Veterinary practitioners should be aware of the potential impact of inherited defects and be prepared to investigate and report animals exhibiting abnormal characteristics. This study provides an example of a dominant acting *de novo* germline mutation associated with a novel lethal phenotype in cattle and illustrates that spontaneous mutations are a risk in cattle breeding. The study reports an *FGFR2* missense mutation as most likely causative mutation supported by striking similarities to *FGFR2* associated syndromic phenotypes in people.

## Additional files

Additional file 1: Overview of Holstein calves submitted for necropsy and genetic analysis. (PDF 178 kb)

Additional file 2: Comparison between a case of the facial dysplasia syndrome (*a* and *c*) and a normal calf (*b* and *d*). *a* and *b*: Longitudinal section through the midline of the head; *c* and *d*: Radiograph of the specimens displayed in *a* and *b*, respectively. *a*-*d*: Bar = 5 cm. (PDF 132 kb)

Additional file 3: Brain lesions in two cases of the facial dysplasia syndrome. *a*: The cerebrum is of reduced size and pathologically lobulated. A diverticulum extending from the left lateral ventricle to the brain surface is externally only covered by the leptomeninges (*arrow*). The cerebellum (*arrowheads*) is of abnormal shape due to compression and dislocated caudally. Ethanol fixed specimen. Bar = 5 cm. *b*: Cross section of the cerebral hemispheres at two levels displaying dilation and abnormally shaped lateral ventricles (*v*) (hydrocephalus). This lesion is associated with atrophy of the periventricular parenchyma and development of diverticula (*) extending to the dorsal surface of the hemispheres. Formalin fixed specimen. Bar = 5 cm. (PDF 213 kb)

Additional file 4: Non-parametric multipoint linkage analysis output data. Z-mean values and LOD scores and their chromosome-wide error probabilities (*P*) along a grid of equally spaced locations (2 Mb) of all 29 autosomes. The minimum and maximum achievable values in the present linkage analyses are given in the first two rows. Chromosome-wide significant Z-mean values and LOD scores and their *P*-values are highlighted in green. (XLSX 53 kb)

Additional file 5: Private *de novo* sequence variants of the sequenced facial dysplasia syndrome affected calf. A total of 500 sequence variants which were absent from the parental genomes. Both variants located in one of the three linked genomic regions are highlighted in green. (XLSX 44 kb)

Additional file 6: An intronic SNP of *DMBT1* linked with facial dysplasia syndrome (FDS) in a family of Holstein cattle. **a** *.* Pedigree drawing and *DMBT1* SNP genotypes. Filled black symbols represent affected calves with FDS, open symbols represent unaffected parents, squares indicate males, and circles indicate females. The case-parent trio subjected to whole genome re-sequencing is indicated by IGV screenshots showing the presence of the chromosome 26 g. 42'862'507G>A *de novo* variant. Note that the electropherograms presented below the pedigree symbols show that the mutant A allele is present in heterozygous form in FDS affected offspring only. **b** *.* Screenshot of the UCSC genome browser illustrating the genomic location (red line) of the *de novo* variant located in an intron of *in silico* predicted *DMBT1* transcripts. (PDF 257 kb)

## Abbreviations

BAM: Binary version of a sequence alignment/map (SAM) file
bp: Base pairs
DMBT1: Deleted in malignant brain tumors 1
EDTA: Ethylenediaminetraacetic acid
FDS: Facial dysplasia syndrome
FGFR2: Fibroblast growth factor receptor 2
GD: Gestation day
IBD: identical by descent
indel: Insertion-deletion
LOD: Logarithm of the odds
Mb: Mega base pairs
OMIA: Online Mendelian Inheritance in Animals
OMIM: Online Mendelian Inheritance in Man
PCR: Polymerase chain reaction
SNP: Single nucleotide polymorphism
UCSC: University of California Santa Cruz
