# Supplementary material for: A de novo missense mutation of FGFR2 causes facial dysplasia syndrome in Holstein cattle
Source: BMC Genet. 2017 Aug 2;18:74. doi: 10.1186/s12863-017-0541-3 (PMC5541750; doi:10.1186/s12863-017-0541-3)
Supplement: Supplementary file 1 — Overview of Holstein calves submitted for necropsy and genetic analysis. (PDF 178 kb) [file 12863_2017_541_MOESM1_ESM.pdf]

Additional file 1. Overview of Holstein calves submitted for necropsy and genetic analysis.

Case	Material	Gender	Gestation age (days)	Body weight (kg)	Histopathology performed
1	Carcass	Female	264	30.0	Yes
2	Carcass	Female	278	26.5	Yes
3	Carcass	Female	281	31.5	Yes
4	Carcass	Female	267	25.5	Yes
5	Carcass	Female	263	25.0	No
6	Head	Unknown	219	Unknown	No
7	Head	Female	277	Unknown	No
