# Supplementary material for: A de novo missense mutation of FGFR2 causes facial dysplasia syndrome in Holstein cattle
Source: BMC Genet. 2017 Aug 2;18:74. doi: 10.1186/s12863-017-0541-3 (PMC5541750; doi:10.1186/s12863-017-0541-3)

Additional file 2. Comparison between a case of the facial dysplasia syndrome (*a* and *c*) and a normal calf (*b* and *d*).

a and *b*: Longitudinal section through the midline of the head; *c* and *d*: Radiograph of the specimens displayed in *a* and *b*, respectively. *a*-*d*: bar = 5 cm.

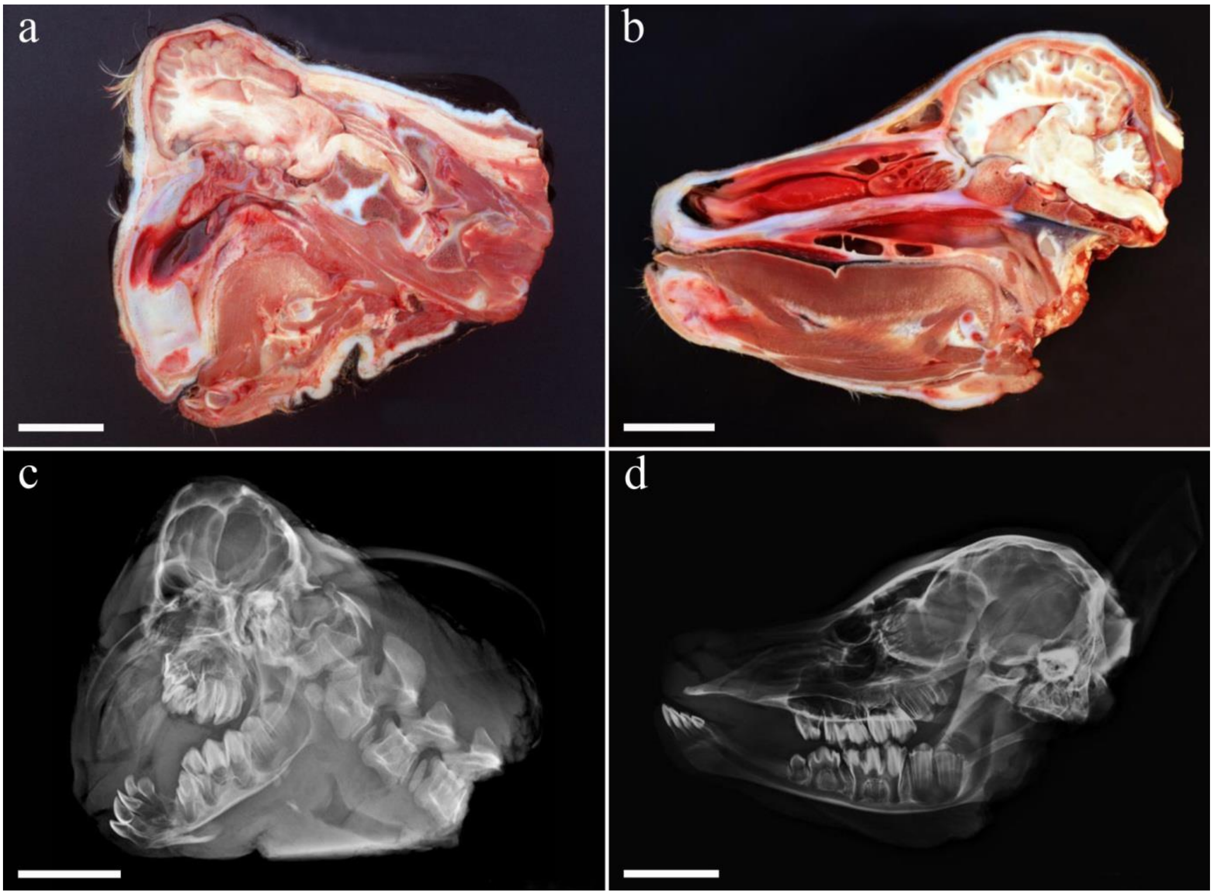

Supplement: Supplementary file 2 — Comparison between a case of the facial dysplasia syndrome (a and c) and a normal calf (b and d). a and b: Longitudinal section through the midline of the head; c and d: Radiograph of the specimens displayed in a and b, respectively. a-d: Bar = 5 cm. (PDF 132 kb) [file 12863_2017_541_MOESM2_ESM.pdf]
