# Supplementary material for: A de novo missense mutation of FGFR2 causes facial dysplasia syndrome in Holstein cattle
Source: BMC Genet. 2017 Aug 2;18:74. doi: 10.1186/s12863-017-0541-3 (PMC5541750; doi:10.1186/s12863-017-0541-3)
Supplement: Supplementary file 3 — Brain lesions in two cases of the facial dysplasia syndrome. a: The cerebrum is of reduced size and pathologically lobulated. A diverticulum extending from the left lateral ventricle to the brain surface is externally only covered by the leptomeninges (arrow). The cerebellum (arrowheads) is of abnormal shape due to compression and dislocated caudally. Ethanol fixed specimen. Bar = 5 cm. b: Cross section of the cerebral hemispheres at two levels displaying dilation and abnormally shaped lateral ventricles (v) (hydrocephalus). This lesion is associated with atrophy of the periventricular parenchyma and development of diverticula (*) extending to the dorsal surface of the hemispheres. Formalin fixed specimen. Bar = 5 cm. (PDF 213 kb) [file 12863_2017_541_MOESM3_ESM.pdf]

Additional file 3. Brain lesions in two cases of the facial dysplasia syndrome.

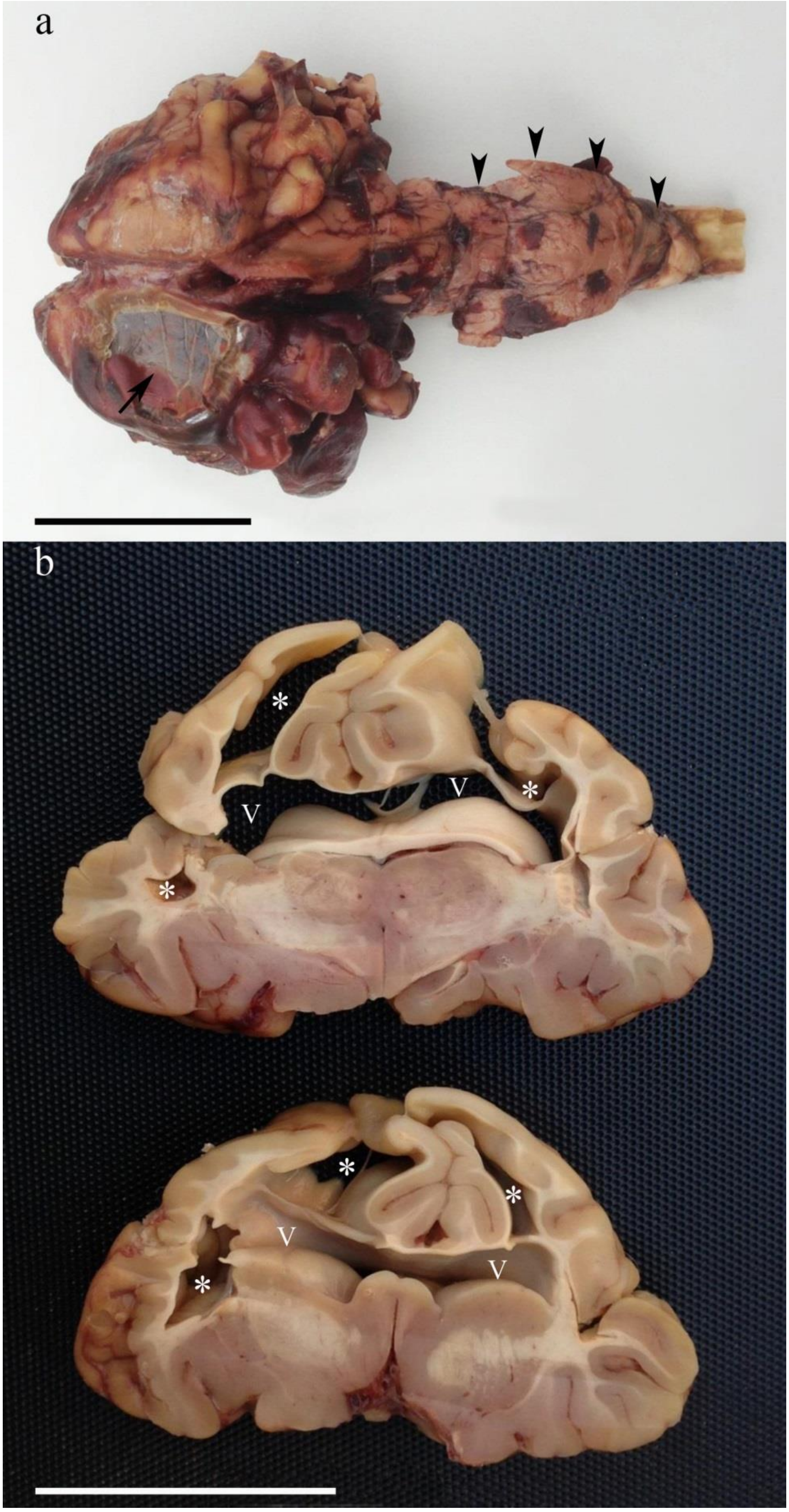

a: The cerebrum is of reduced size and pathologically lobulated. A diverticulum extending from the left lateral ventricle to the brain surface is externally only covered by the leptomeninges (*arrow*). The cerebellum (*arrowheads*) is of abnormal shape due to compression and dislocated caudally. Ethanol fixed specimen. Bar = 5 cm.

b: Cross section of the cerebral hemispheres at two levels displaying dilation and abnormally shaped lateral ventricles (*v*) (hydrocephalus). This lesion is associated with atrophy of the periventricular parenchyma and development of diverticula (*) extending to the dorsal surface of the hemispheres. Formalin fixed specimen. Bar = 5 cm.
