# Supplementary material for: A de novo missense mutation of FGFR2 causes facial dysplasia syndrome in Holstein cattle
Source: BMC Genet. 2017 Aug 2;18:74. doi: 10.1186/s12863-017-0541-3 (PMC5541750; doi:10.1186/s12863-017-0541-3)

Additional file 6. An intronic SNP of *DMBT1* linked with facial dysplasia syndrome (FDS) in a family of Holstein cattle.

a. Pedigree illustration and *DMBT1* SNP genotypes. Filled black symbols represent calves affected by FDS, open symbols represent unaffected parents, squares indicate males, and circles indicate females. The case-parent trio subjected to whole genome re-sequencing is indicated by IGV screenshots showing the presence of the chromosome 26 g. 42'862'507G>A *de novo* variant. Note that the electropherograms presented below the pedigree symbols show that the mutant A allele is present in heterozygous form in FDS affected offspring only.

b. Screenshot of the UCSC genome browser illustrating the genomic location (*red line*) of the *de novo* variant located in an intron of *in silico* predicted *DMBT1* transcripts.

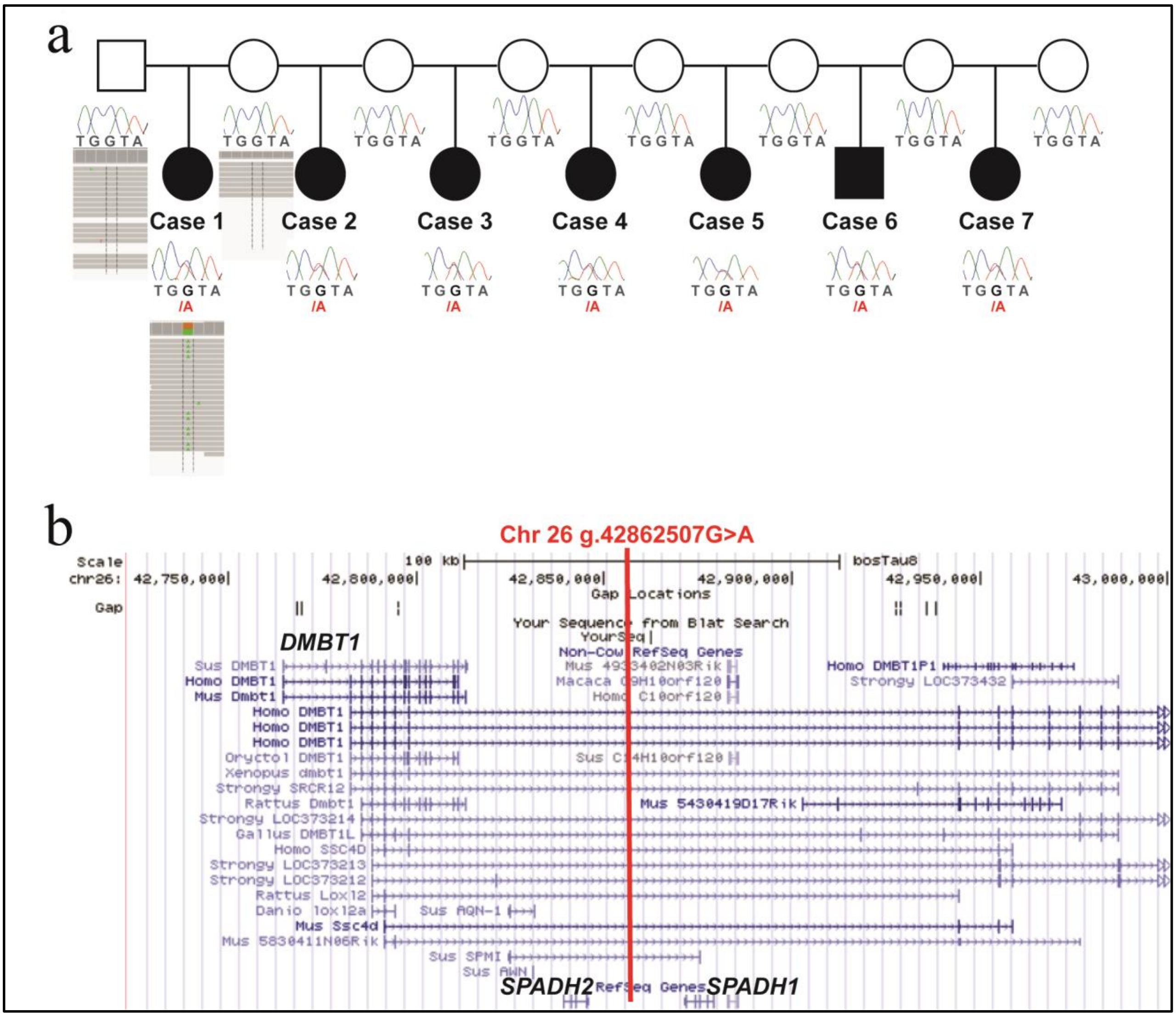

Supplement: Supplementary file 6 — An intronic SNP of DMBT1 linked with facial dysplasia syndrome (FDS) in a family of Holstein cattle. a . Pedigree drawing and DMBT1 SNP genotypes. Filled black symbols represent affected calves with FDS, open symbols represent unaffected parents, squares indicate males, and circles indicate females. The case-parent trio subjected to whole genome re-sequencing is indicated by IGV screenshots showing the presence of the chromosome 26 g. 42'862'507G>A de novo variant. Note that the electropherograms presented below the pedigree symbols show that the mutant A allele is present in heterozygous form in FDS affected offspring only. b . Screenshot of the UCSC genome browser illustrating the genomic location (red line) of the de novo variant located in an intron of in silico predicted DMBT1 transcripts. (PDF 257 kb) [file 12863_2017_541_MOESM6_ESM.pdf]
